# Optothermal dynamics in whispering-gallery microresonators

**DOI:** 10.1038/s41377-019-0239-6

**Published:** 2020-02-24

**Authors:** Xuefeng Jiang, Lan Yang

**Affiliations:** Department of Electrical and System Engineering, Washington University in St. Louis, St. Louis, MO 63130 USA

**Keywords:** Microresonators, Nonlinear optics, Photonic devices

## Abstract

Optical whispering-gallery-mode microresonators with ultrahigh quality factors and small mode volumes have played an important role in modern physics. They have been demonstrated as a diverse platform for a wide range of applications in photonics, such as nonlinear optics, optomechanics, quantum optics, and information processing. Thermal behaviors induced by power build-up in the resonators or environmental perturbations are ubiquitous in high-quality-factor whispering-gallery-mode resonators and have played an important role in their operation for various applications. In this review, we discuss the mechanisms of laser-field-induced thermal nonlinear effects, including thermal bistability and thermal oscillation. With the help of the thermal bistability effect, optothermal spectroscopy and optical nonreciprocity have been demonstrated. By tuning the temperature of the environment, the resonant mode frequency will shift, which can also be used for thermal sensing/tuning applications. The thermal locking technique and thermal imaging mechanisms are discussed briefly. Finally, we review some techniques employed to achieve thermal stability in a high-quality-factor resonator system.

## Optical microresonators: Investigating light’s tiny whispers

Optical whispering-gallery mode microresonators can trap light in a highly confined volume, similar to the behavior of sound waves in the famous whispering gallery of St Paul’s Cathedral, London, and are being used for a diverse variety of optical studies and applications. Xuefeng Jiang and Lan Yang at Washington University in St. Louis, Missouri, USA, review the optothermal behaviors induced by power build-up in the resonators or environmental perturbations in these systems. Several techniques have been developed with the help of optothermal behaviors, such as optothermal spectroscopy, thermal tuning, thermal locking, thermal imaging, etc. Temperature fluctuations affect the resonant frequencies of the light they contain, which can be used for thermal sensing applications, but must also be controlled to avoid problems caused by heat fluctuations when the microresonators are used in other applications.

## Introduction

In the last two decades, whispering-gallery-mode (WGM) microresonators^1^ have enabled numerous advances in fundamental science and technology, including optomechanics, non-Hermitian physics, communications, frequency combs, high-performance sensors, and cavity quantum electrodynamics (QED)^2–22^. The capability of WGM microresonators to trap light in a highly confined volume for a long period of time significantly enhances light−matter interactions and enables a high-power build-up^23,24^. Consequently, thermal effects and the associated dynamics are ubiquitous in WGM microresonators. For example, thermo-optic nonlinear dynamics^25–28^ and thermal instability^29,30^ have been observed in various applications. Specifically, temperature fluctuations affect the material refractive index and/or size of the resonator, both of which modify the mode distributions and shift the resonance frequencies of WGMs. Stable and continuous operation is critical for many applications, such as bio/chemical sensing^31–39^, optomechanics^18,40–42^, microlasers^43–47^, non-Hermitian physics^19^, and nonlinear photonics^2,3,48–54^. Taking WGM sensing as an example, the resonance shifts induced by a target of interest^34^ are typically mixed with the thermally induced mode shift. Therefore, various thermal-stability techniques have been developed to suppress thermal noise in WGM microresonator applications, such as sensing and metrology, where thermally induced signal fluctuations are undesired. In addition, by making use of the thermal effects, innovative photonic techniques have been developed. For example, the thermal tuning technique has been applied to adjust the resonator frequency in parity-time (PT) symmetric resonator systems^19,55,56^ and to accurately measure the optothermal properties of resonators^57,58^. Furthermore, thermal sensing applications have also been demonstrated in many WGM microresonators using the thermo-optic effect and/or the thermal expansion effect. In addition, the thermal scanning technique has been demonstrated to reduce the noise of a frequency combs and optical solitons^59^. Moreover, thermal locking^60,61^ and thermal imaging techniques^62–64^ have also been developed by taking advantage of thermal instability and thermal absorption, respectively, which will find broad applications in sensing, microscopy, and spectroscopy.

Here, in this review, the physical mechanism of the thermal nonlinear effects induced by probe/pump laser fields will first be discussed, including both the thermal bistability in a microresonator with a uniform material composition and the thermal oscillation in a hybrid-material microresonator. Furthermore, optothermal spectroscopy, a thermal relaxation parameter measurement, and optical thermal nonreciprocity will also be introduced as applications of the thermal bistability effect. Second, thermo-optic applications, including thermal tuning, thermal scanning, thermal sensing, thermal locking and photothermal imaging techniques, will be discussed. Third, we will review some techniques to realize thermal stability. Finally, we offer a brief summary of thermo-optic dynamics and techniques in WGM microresonators.

## Thermal nonlinear effects

Ultrahigh power build-up benefitting from a high-quality factor (*Q*) and small mode volume (*V*) significantly enhances the absorption-induced thermal nonlinearity in WGM microresonators. Thermal bistability behavior typically occurs when scanning across a high-*Q* mode, which is translated into linewidth broadening/narrowing behavior^26^. In addition, a thermal oscillation may arise in a microresonator made of hybrid materials with different temperature coefficients.

### Thermal bistability

In a WGM microresonator, the resonance frequency/wavelength response to changes in temperature is affected by both the thermo-optic and thermal expansion effects. The thermo-optic effect (d*n*/d*T*) represents the temperature dependence of the material refractive index, while the thermal expansion effect transfers temperature fluctuations into changes in the cavity size. Both the cavity size and refractive index can affect the resonance frequency according to the WGM resonance condition (2*πn*(*T*)*R*(*T*) = *mλ*(*Τ*)). The temperature of a WGM resonator can be tuned by an external thermal source or heat generated from optical absorption. External thermal sources have been widely applied to resonance frequency tuning and thermal sensing, which will be discussed later. In this section, we focus on the temperature change induced by the optical field itself. The resonance wavelength shift (Δ*λ*) as a function of a temperature change (Δ*T*) can be expressed as

1 $${\mathrm{\Delta }}\lambda \left( {{\mathrm{\Delta }}T} \right) = \lambda _0\left( {\frac{1}{n}\frac{{{\mathrm d}n}}{{{\mathrm d}T}} + \frac{1}{D}\frac{{{\mathrm d}D}}{{{\mathrm d}T}}} \right){\mathrm{\Delta }}T$$

where *λ*_0_, *D* and *n* are the resonance wavelength, effective diameter and refractive index of the cold cavity, respectively. d*n*/d*T* and (1/*D*)(d*D*/d*T*) represent the coefficients of the thermal refraction and thermal expansion of the cavity, respectively.

The temperature dynamics can be described by^26^,

2 $$C_P{\mathrm{\Delta }}\dot T\left( t \right) = I\frac{1}{{\left( {\frac{{\lambda _p - \lambda _0(1 + \alpha {\mathrm{\Delta }}T)}}{{{\updelta }}\lambda /2}} \right)^2 \,+\, 1}} - K{\mathrm{\Delta }}T\left( t \right)$$

where *C*_*p*_ and *K* are the effective heat capacity and effective thermal conductivity of the cavity material, respectively. *I* represents the optical power that actually heats the cavity, and δ*λ* is the mode linewidth of the cold cavity. The first term of Eq. (2) represents the heat generated from the resonant mode, while the second term represents the heat transfer to the environment.

**Fig. 1:**
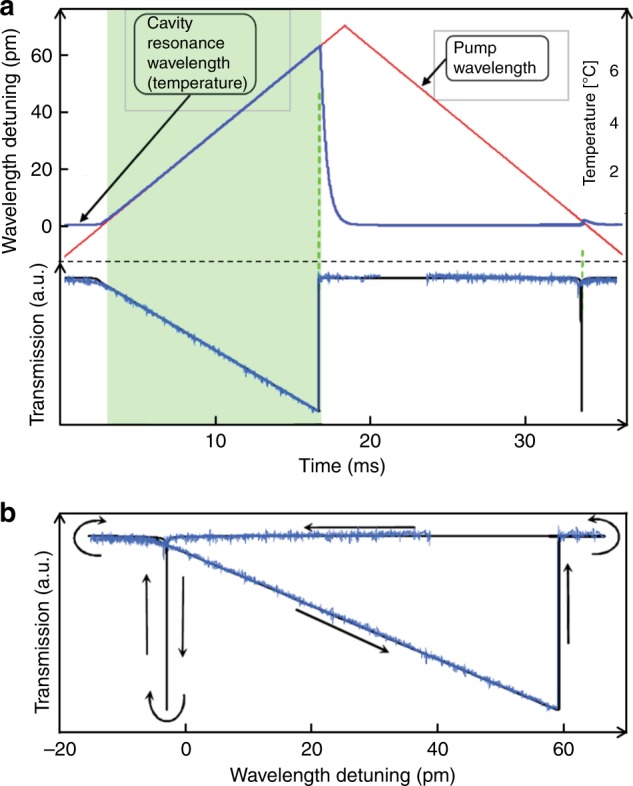
Dynamic thermal bistability in a silica microtoroid resonator^26^. **a** Calculated transmission spectrum, cavity temperature, and resonance wavelength and the measured transmission spectrum during the wavelength up- and downscan processes. **b** Repeating transmission as a function of wavelength detuning in the upscanning (broadening) and downscanning (narrowing) processes. Reprinted with permission from ref. ^26^ [OSA The Optical Society]

The transmission spectrum of a silica microresonator appears as a triangular shape and a sharp dip during the wavelength up- and downscanning processes, known as thermal broadening and narrowing, respectively. Figure 1 shows the transmission, temperature, and resonance shift of a silica toroidal microresonator during the wavelength up- and downscanning processes^26^. Taking the wavelength upscanning process as an example, as the wavelength of the pump laser approaches the resonance wavelength (at *t* ~ 2 ms), the cavity begins to heat up, which redshifts the resonance wavelength, making the upscan a “pursuit process” between the resonance wavelength and scanning pump wavelength; i.e., both the resonance wavelength and scanning pump wavelength shift in the same direction. Specifically, the pump follows the moving resonance wavelength, and the detuning between them decreases linearly; therefore, more pump is coupled into the resonator as the upscan proceeds. The pursuit process continues until the resonant point is caught up by the pump wavelength (*t* ~ 16 ms). At this point, the thermal absorption is maximal, and the compensation of the heat dissipation to the environment by thermal absorption can no longer be maintained. Beyond this point, the resonant state is rapidly lost, since the pump laser cannot shift the resonance wavelength further. On the other hand, in the case of the wavelength downscanning process, a narrowed resonance lineshape is observed due to an opposite process between the pump and resonance wavelengths; i.e., the thermally induced resonance shift and the scanning pump wavelength move in opposite directions, which results in passing through the resonance quickly.

**Fig. 2:**
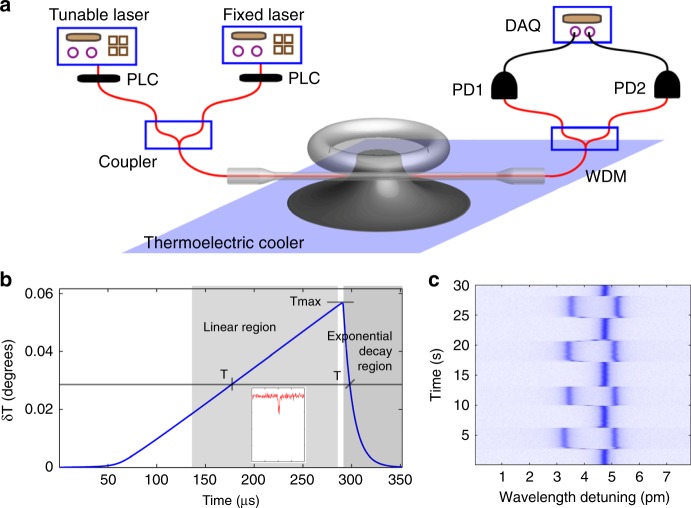
Optothermal resonance scanning spectroscopy. **a** Schematics of the setup for optothermal spectroscopy. PLC polarization controller, PD photodetector, PM power meter, DAQ data acquisition, WDM wavelength division multiplexer. **b** Calculated temperature change of a high-*Q* mode during the wavelength upscanning process. Inset: transmission of a high-*Q* mode in the 1550 nm wavelength band scanned by a thermal pump WGM in the 1440 nm wavelength band^50^. **c** Transmission spectrum frames acquired by optothermal spectroscopy. Here, a nanofiber tip is repeatedly approaching and moving away from the microtoroid^57^. Reprinted with permission from ref. ^57^ [American Institute of Physics]

As an application, thermal bistability can be used to scan the temperature of a WGM resonator during the wavelength upscanning process^57^. There is a linear region in Fig. 1a, where the temperature of the cavity increases linearly with time. This property can be used to thermally scan WGMs with a fixed-wavelength laser (Fig. 2b). Specifically, a high-power tunable laser in the 1440 nm wavelength band is used to pump and scan the temperature of the microresonator, while another fixed-wavelength laser in the 1550 nm wavelength band is used to probe a high-*Q* WGM when the resonance wavelength is thermally scanned. This thermal-bistability-assisted scanning scheme enables stable optothermal spectroscopy by removing thermal-noise-induced spectral perturbations, which could extend WGM applications to any wavelength band. Furthermore, optothermal spectroscopy has been used to control transmission spectra and optical gains in both erbium-doped^65^ and Raman^66^ WGM microlasers.

Recently, the thermal relaxation time and effective thermal conductance of a WGM could also be estimated by fitting two nearby optical modes modulated by the thermal effect^58,67^. Specifically, two nearby WGMs of a toroidal microresonator are scanned by a weak probe laser and a strong probe laser, where the weak probe records the wavelength detuning of the two WGMs; thus, the thermal relaxation time could be derived from the change in the detuning in the case of a strong probe. By tuning the strong probe power, the quantified thermal relaxation process could be fitted.

Optical nonreciprocal devices have attracted increasing attention in the past few years^68^. The standard method to realize optical nonreciprocity is through the magneto-optical effect, which requires an external magnetic field. Magnetic-free optical nonreciprocity in microresonators based on thermal nonlinear effects was proposed by Fan et al.^69^ Specifically, a silicon microring resonator coupled with two waveguides forms an add-drop filter system. The coupling strengths of these two waveguides are distinct due to the different ring-waveguide gaps. When exciting the resonator in opposite directions, the resonator undergoes different thermal broadenings in the transmission spectra. When choosing the resonant point as the operating wavelength, a nonreciprocal transmission ratio up to 40 dB could be achieved with this add-drop filter due to the thermal nonlinear effect.

### Thermal oscillation

**Fig. 3:**
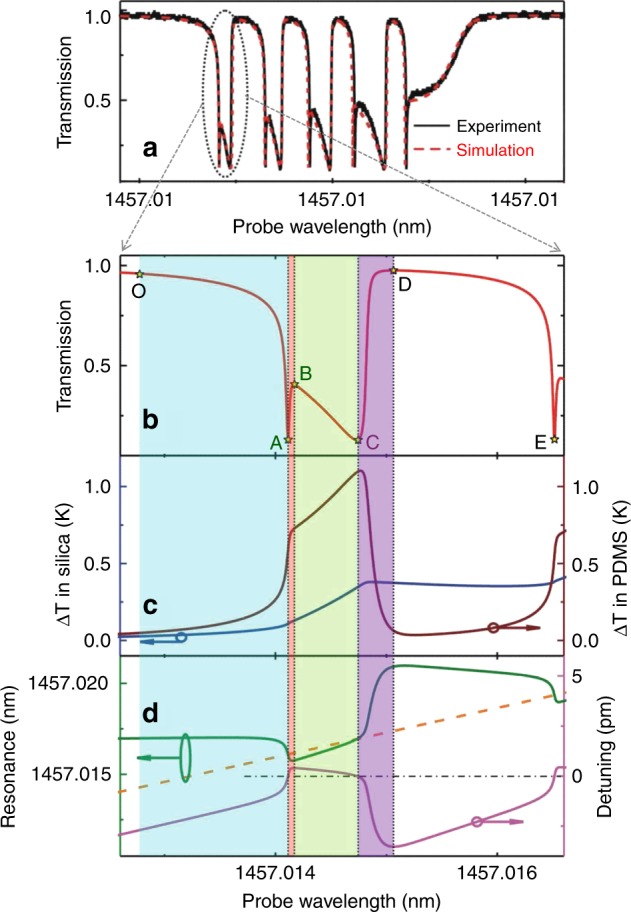
Thermal oscillations in a PDMS-coated silica microtoroid during the wavelength upscanning process. **a** Measured and simulated optical transmission spectra. **b** Simulated transmission oscillation spectrum. **c** Simulated temperature dynamics in both silica and PDMS. **d** Calculated resonance wavelength (*λ*_r_), probe wavelength (*λ*_p_), and the associated variation in the detuning. Zero detuning is marked by a dot-dashed line (black) in (**d**)^70^. Reprinted with permission from ref. ^70^ [OSA The Optical Society]

Hybrid microresonators can produce novel dynamic properties, such as thermal oscillation. Here, we discuss this phenomenon with a polydimethylsiloxane (PDMS)-coated microtoroid as an example^70^. Figure 3 shows the experimental results and numerical simulations of dynamic changes in the transmission (Fig. 3b), temperature fluctuations in both the silica and PDMS layers (Fig. 3c), and the resonance wavelength in the wavelength upscanning process (Fig. 3d). There are four thermodynamic processes in the transmission spectrum, marked by regions with different colors.

#### Region I

From point O to A in Fig. 3b, the probe laser is coupled into the microresonator gradually when the probe wavelength (*λ*_p_) approaches the resonance wavelength (*λ*_r_). The temperature of the microresonator increases during this process due to thermal absorption, leading to a refractive index decrease/increase within the mode volumes in the PDMS/silica. As shown in Fig. 3c, the temperature increase in the PDMS layer is much larger than that in the silica layer, because the absorption coefficient of PDMS is much larger than that of silica in this wavelength band. Thus, the resonance is dominated by the thermo-optic effect of the PDMS layer, exhibiting a blueshift (green curve in Fig. 3d). On the other hand, considering that *λ*_p_ and *λ*_r_ shift in opposite directions, their detuning (Δ*λ*) decreases rapidly (purple curve in Fig. 3d), which gives rise to a rapid decrease in the transmission spectrum. Point A is the exact resonance frequency of the WGM (Δ*λ* = 0), at which point the transmission decreases to the minimum value.

#### Region II

From point A to B, the temperatures in both layers continue to increase. The resonance wavelength *λ*_r_ exhibits a blueshift at the beginning due to the dominance of the PDMS. Considering that *λ*_p_ is larger than *λ*_r_ at point A, the transmission increases rapidly with increasing Δ*λ* (Fig. 3b, d). The influence of the thermo-optic effect in the PDMS decreases as the optical absorption decreases, and *λ*_r_ undergoes a transition process from a blueshift to a redshift in this region. Although the resonance wavelength *λ*_r_ moves in the same direction as the wavelength upscanning process after the transition point, the speed of the redshift of *λ*_r_ cannot catch up with the wavelength upscanning process; thus, the transmission continues to increase. Note that the speed of the redshift of *λ*_r_ increases gradually. Finally, at point B, the speed of the redshift of *λ*_r_ is the same as that of scanning *λ*_p_. As a result, at point B, both the transmission and detuning Δ*λ* reach a local maximum point.

#### Region III

From point B to C, the thermo-optic effect in silica dominates due to the stable temperature increase in the silica section of the mode volume. Therefore, the speed of the redshift of *λ*_r_ is much faster than the speed for *λ*_p_, resulting in a decrease in both Δ*λ* and the transmission. At point C, *λ*_r_ equals *λ*_p_; thus, the transmission value reaches the local minimum a second time because the optical mode is on-resonance again.

#### Region IV

From point C to D, the redshift of *λ*_r_ increases continually, resulting in an increase in both Δ*λ* and the transmission. Thus, the optical-absorption-induced heat decreases. When the optical absorption is smaller than the dissipation to the environment, the temperature in the mode volume starts to decrease. The temperature decreasing speed in PDMS is faster than the speed in silica, because the heat dissipation in PDMS is much larger. Thus, the dynamic behavior of *λ*_r_ is dominated by the thermo-optic effect of PDMS (green curve in Fig. 3d). The transmission increases quickly due to the fast separation of *λ*_r_ from *λ*_p_. Point D represents the nonresonant point, where the wavelength detuning is large enough, and thus the transmission returns to the maximum. After passing through point D, a similar cycle repeats the whole process (O−A−B−C−D), which gives rise to the thermal oscillation with a period from O to D.

Thermal oscillations have been experimentally realized in not only microresonators with a coating, such as a PDMS-coated microtoroid^70^, PDMS-coated microsphere^71^, and PMMA-coated microtoroid^72^, but also uncoated microresonators with a uniform material composition such as a silica microsphere^73,74^, ZBLAN (ZrF4-BaF2-LaF3-AlF3-NaF) microsphere^75^, millimeter-sized BaF_2_ disk^76^, silicon nitride^77^, and lithium-niobate^78,79^ microdisk. The mechanisms of thermal dynamics in uncoated resonators are slightly different from those of coated resonators. They all result from the interplay of multiple effects with different influences on the resonance condition. For example, the thermal oscillation in a silica microsphere originates from the interplay between the Kerr nonlinear effect and the thermo-optic effect near 20 K^74^; the thermal oscillation in a ZBLAN microsphere is based on the interplay of three effects, including the Kerr effect, the thermo-optic effect, and thermal expansion^75^; the oscillation in a BaF_2_ disk is due to the interactions of a positive thermo-optic effect, a negative thermoelastic effect, and the intrinsic Kerr nonlinearity^76^; and the oscillations in Si_3_N_4_ and LiNbO_3_ microdisks result from the interplay of two nonlinear effects, i.e., a fast thermo-optic nonlinearity and a slow process, such as a thermomechanical nonlinearity^77^, heat dissipation process^78^ or photorefractive effect^79^. It should be noted that the frequency of the thermal oscillation is typically on the order of Hz to kHz. However, Luo et al. report a MHz-level thermal oscillation in a PDMS-coated microsphere, which was considered to result from the competition between the thermo-optic effect and thermal-expansion effect of PDMS^71^.

## Thermo-optic applications

### Thermo-optic tuning

Tuning a WGM resonance is critical in many applications, such as PT symmetry^19^, tunable microlasers^80–82^, optical filters^83^, and cavity QED^84,85^. Various frequency tuning techniques have been explored, such as thermal^86,87^, pressure/strain^80–82,85,88–90^, electro-optical^91,92^, magnetic-field^93^, electrothermal^94^, internal aerostatic pressure^95,96^, chemical etching^97^, and laser polishing^98^ techniques. Every technique has its own limitation. Specifically, electro-optical and electrothermal techniques are only applicable to special materials; pressure/strain and internal aerostatic pressure techniques are suitable for particular resonator structures, such as microbubbles; and etching and laser polishing techniques are disruptive, leading to permanent changes in the physical structures of the resonators. Among these techniques, thermal tuning is the simplest technique and essentially applies to all resonator structures/materials.

**Fig. 4:**
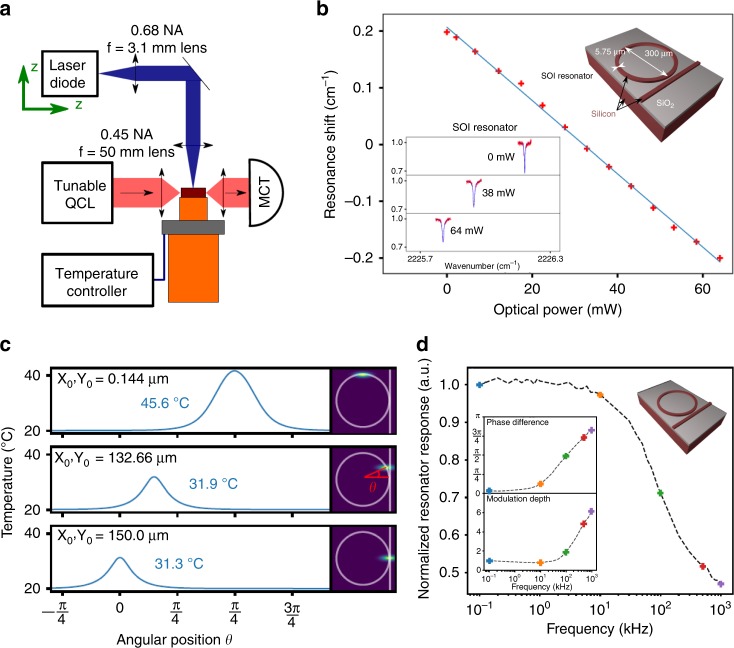
Direct thermo-optic tuning of a silicon microresonator. **a** Schematic of the setup employed for direct thermo-optic tuning. **b** The resonance frequency is shifted while adjusting the optical power of the laser diode that serves as the pump to drive the thermo-optic effect. Bottom-left inset: the transmission signal at different optical powers, showing the resonance shift. Top-right inset: schematic of the device. **c** For a focused beam with dimensions of *w*_*x*_ ~ 40 μm and *w*_*y*_ ~ 13 μm, the temperature profile along the angular position is plotted for three different beam positions. **d** The normalized response of the resonator as a function of the modulation frequency. Inset: the phase difference between the laser power and the detected mid-IR signal and the modulation depth of the mid-IR signal^99^. Reprinted with permission from ref. ^99^ [OSA The Optical Society]

As an example, here we discuss a direct tuning method for a silicon microresonator that employs the thermo-optic effect introduced by a visible laser diode^99^. As shown in Fig. 4a, the laser diode beam is focused on the top surface of the silicon microresonator. The resonance frequency shift as a function of the laser diode power is shown in Fig. 4b, which demonstrates a tuning rate of 0.0067 cm^−1^/mW. It is worth noting that this direct thermo-optic tuning is a local and noninvasive method. In Fig. 4c, the temperature along the resonator depends on the angle *θ* for different pump positions, which affects the overlap of the laser beam with the resonant mode and leads to changes in the thermo-optic effect. Note that the local temperature when pumping at *θ* = *π*/2 is much larger than the temperature of the other two positions; this is attributed to the elliptic profile of the focused beam. In addition, the direct tuning method promises a much faster response than traditional thermal tuning methods. The normalized resonator response in Fig. 4d, defined as the normalized product of the pump power and the transmitted signal, is plotted as a function of the modulation frequency with a cut-off frequency of approximately 10 kHz. In the inset, the phase difference between the laser power and the transmitted light is presented, and the modulation depth of the signal as a function of the modulation frequency is also plotted.

### Thermal scanning for comb and soliton applications

Frequency comb generation and optical solitons, originating from cascaded four-wave mixing in a WGM microresonator, have attracted increasing interest in the past decade^2,100–108^. The generation of both a frequency comb and soliton typically relies on a tunable laser source, which has intrinsic drawbacks. Specifically, the linewidths of the best tunable lasers are usually on the order of 100 kHz, which is much broader than the linewidth of the best single-frequency laser. The broad linewidth and noisy amplitude of the tunable laser used for pumping limit the performance of the comb. In contrast, a single-frequency laser can provide much lower noise and a narrower linewidth than tunable lasers by using a reference frequency locking technique. For example, a narrow linewidth of < 40 mHz has been demonstrated in a locked single-frequency laser^109^, which could be used as the pump source of a comb to reduce the noise on the generated comb lines.

**Fig. 5:**
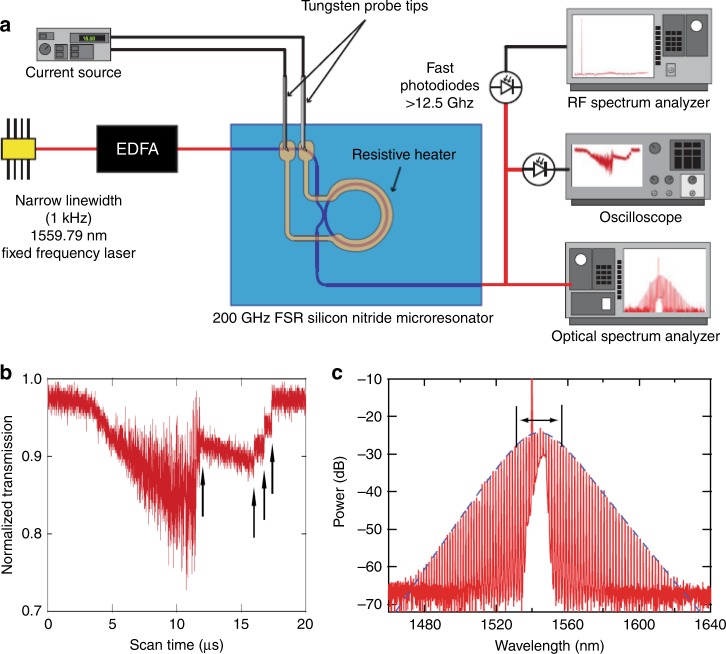
Thermal scanning technique for soliton applications. **a** Schematics of the setup employed for the generation and characterization of optical solitons with the thermal scanning method. **b** Oscilloscope trace of the pump transmission as the current of the integrated heater is modulated with a triangular waveform. The steps indicated by the arrows are characteristic of transitions between different multisoliton states. **c** Measured optical spectrum for a single-soliton mode-locked state with the fitted sech^2^-pulse spectrum (blue dashed line)^59^. Reprinted with permission from ref. ^59^ [OSA The Optical Society]

Joshi et al. demonstrated a frequency comb and mode-locked soliton with a single-frequency pump laser by thermally scanning the resonance frequency of the microresonator^59^. As shown in Fig. 5a, an integrated platinum resistive microheater is fabricated on top of an oxide-clad Si_3_N_4_ resonator. The resonance frequency could be controlled by tuning the current of the integrated microheater due to the refractive index change induced by the thermo-optic effect. A single-frequency laser with a linewidth of ~1 kHz is used as the pump laser, which is then amplified by an erbium-doped fiber amplifier (EDFA) and coupled into the on-chip bus waveguide through a fiber lens. The transmitted signals are monitored by a fast photoreceiver (>12.5 GHz) and then analyzed by an optical spectrum analyzer, RF spectrum analyzer, and oscilloscope. By applying a triangular modulation to the heater current, the scanned transmission spectrum measured by the oscilloscope is shown in Fig. 5b, where the step-like structures marked by arrows indicate the transitions into mode-locked soliton states^110–112^. Furthermore, the recorded optical comb spectrum agrees with the fitted sech^2^ pulse spectrum (dashed blue curve in Fig. 5c), corresponding to a mode-locked single-soliton state.

### Thermal sensing

A WGM microresonator can be utilized as a thermal sensor due to its ultrasensitive response to the temperature of the surrounding environment. In a WGM-based thermal sensor, the resonance wavelength/frequency is a linear function of both the refraction index and size of the WGM microresonator, both of which vary with the temperature of the environment due to the thermo-optic and thermal expansion effects, respectively. In principle, a cavity material with a larger thermo-optic coefficient and/or thermal expansion coefficient typically leads to a larger frequency shift and thus allows for a more accurate temperature measurement.

Thermal sensing experiments have been demonstrated using silica- and silicon-based devices^113–115^. To improve the sensitivity of a thermal sensor, one can utilize a microresonator made of materials with larger thermo-optic coefficients, such as PDMS^116,117^, UV-curable adhesives^118^, lithium niobate^119^, and dye-doped photoresists^120^, which give rise to a much better sensitivity. For example, a PDMS microsphere thermal sensor demonstrated a sensitivity of 0.245 nm/K^117^. However, materials with large thermal expansion coefficients, such as silk, have also been used as a WGM thermal sensor. Specifically, a high thermal sensing sensitivity of −1.17 nm/K was realized in a silk fibroin microtoroid, which was attributed to the large thermal expansion effect^121^.

**Fig. 6:**
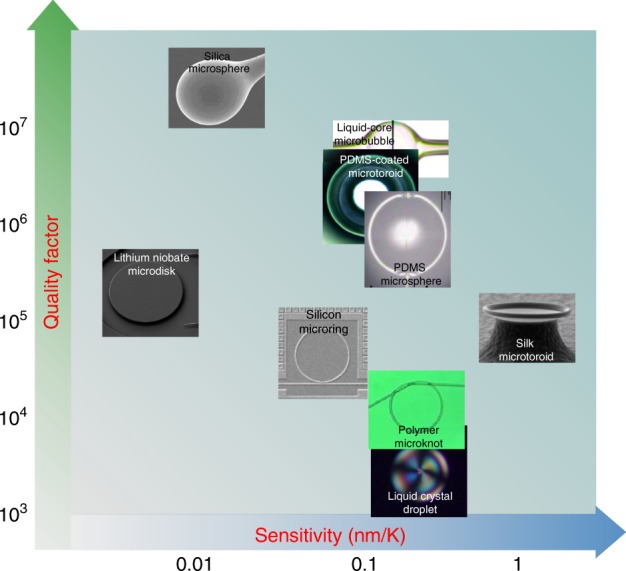
*Q* factor and sensitivity of WGM thermal sensors made of different materials at room temperature

An alternative experimental design for temperature sensing involves the use of microdroplets, which can be made of a variety of materials, such as dye-doped cholesteric liquids, liquid crystals^122^, and oils^123^. The advantages of a microdroplet-based thermal sensor are the ease of integration with conventional microfluidics and diverse choices of materials with relatively high thermal refraction coefficients. Ward et al. also utilized thin-shelled microbubbles filled with air for temperature sensing^124,125^. Figure 6 summarizes the *Q* factors and sensitivity of some typical WGM thermal sensors operating at room temperature. Note that the silk microtoroid thermal sensor demonstrates the highest sensitivity (1.17 nm/K), benefiting from the ultrahigh thermal expansion coefficient of the silk material^121^. It is worth mentioning that lithium niobate is an excellent candidate for self-referenced thermal sensing due to its strong thermo-optic birefringence. A preliminary study demonstrated self-referenced thermal sensing with an on-chip lithium niobate microdisk resonator with *Q* ~ 10^5^ by using the different thermo-optic responses of ordinary and extraordinary light in a birefringent material^119^. Experimentally, millimeter-sized lithium niobate WGM resonators^126^ with *Q* factors up to 10^8^ and chip-based lithium niobate microdisks^127^ with *Q* factors up to 10^7^ have been achieved, paving the way for the development of a high-performance thermal sensor.

### Thermal locking

**Fig. 7:**
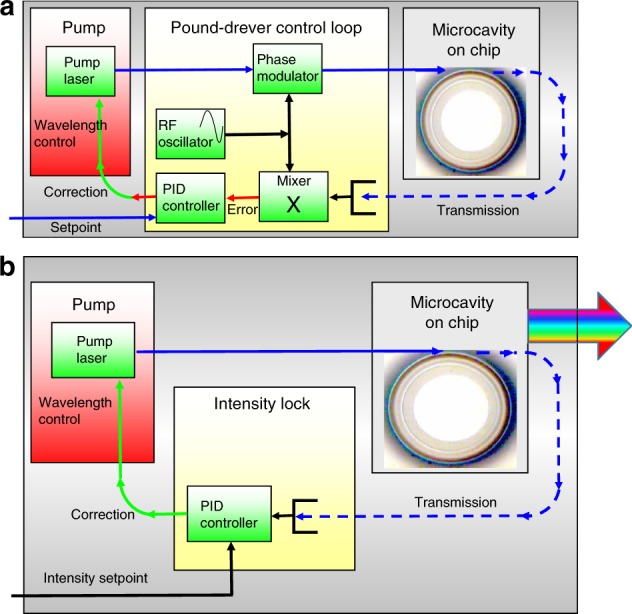
Experimental setups of two locking systems. **a** Pound–Drever–Hall (PDH) locking system. **b** Thermal locking system^60^. Reprinted with permission from ref. ^60^ [OSA The Optical Society]

The Pound–Drever–Hall (PDH) locking technique has been widely used to lock the optical frequency of a probe/pump laser to a desired resonance frequency in various microresonator applications, such as optomechanics, nonlinear optics, and sensing^128^. In this method, a feedback voltage signal is applied to the laser head to lock the probe laser frequency to the resonance frequency or a frequency with a constant detuning from the resonance. As shown in Fig. 7a, a PDH error signal is generated by measuring the phase difference between the transmitted light and the probe laser^60^. A proportional–integral–derivative (PID) controller can read the laser’s offset from the cavity resonance and feed this signal into a servo loop, which adjusts the frequency by minimizing the PDH error signal; the error signal is linear with the frequency detuning near the resonance, making the servo loop straightforward to manage.

Similarly, the thermally modified transmission spectrum during the wavelength upscanning process is nearly a linear function of the wavelength detuning, as shown in Figs. 1a and 2b, which can serve as an error signal for locking. Specifically, the linear region of the transmission function will be used as an input to the PID controller to stabilize the optical power coupled into the resonator, as shown in Fig. 7b. In this linear region, a small pump power increase (decrease) can heat up (cool down) the temperature of the resonator and thus increase (decrease) the frequency detuning by pushing the cavity resonance far away from (close to) the pump laser frequency. As a result, the optical absorption will decrease (increase). The whole process forms a negative feedback loop, which enables thermal locking^60^. It is worth noting that this thermal locking technique is only capable of locking to the resonance slope in the blue detuned region.

However, external environment perturbations may break the thermal locking status, which makes it difficult to achieve long-term locking. Therefore, to solve this problem, McRae et al. proposed another locking mechanism by combining the thermal locking technique with an optical locking technique, which could achieve long-term locking up to 12 h^61^. Specifically, the thermal locking technique is utilized to lock the resonance frequency of the microresonator to the probe laser frequency by optimizing the balance between optical absorption and thermal dissipation. An optical feedback locking system induced by scattering centers is utilized to achieve fast feedback control. The system locks the probe laser frequency to the resonance frequency by maximizing the constructive interference between the intracavity field and the feedback laser field.

### Photothermal absorption spectroscopy and imaging

**Fig. 8:**
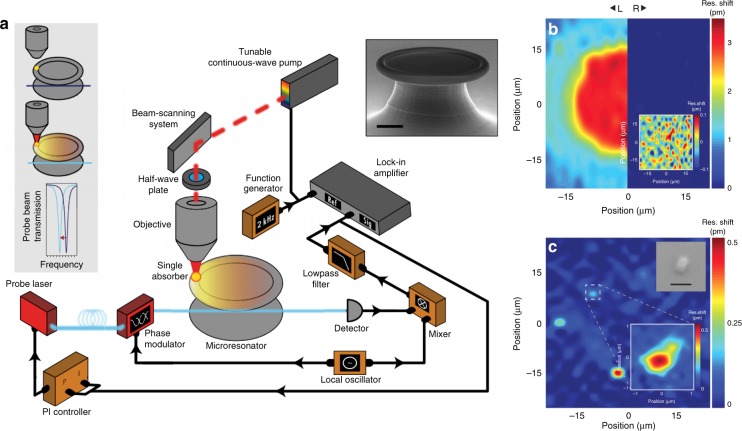
Photothermal absorption spectroscopy and imaging. **a** Schematics of the setup employed for photothermal absorption spectroscopy and imaging. Inset (left): diagram of the thermal shift induced by the photothermal absorption of gold nanoparticles. Inset (right): scanning electron microscopy (SEM) image of a silica microtoroid. Scale bar: 10 μm^62^. **b** Large-area maps of a silica-on-silicon toroid (left) and an all-glass toroid (right). **c** Photothermal imaging of a single gold nanorod on an all-glass microtoroid. Inset (top): SEM image of a nanorod with a scale bar of 100 nm. Inset (bottom): high-resolution photothermal imaging of the nanorod^64^. **a** Reprinted with permission from ref. ^62^ [Nature Publishing Group]; **b**, **c** Reprinted with permission from ref. ^64^ [John Wiley & Sons, Inc.]

Previous studies are based on the thermal dynamics of the whole cavity or materials in the mode volume, while small temperature changes in the local environment of microresonators can also be detected by the thermal shift of the resonance, which is a similar effect used in photothermal absorption spectroscopy^62^. As shown in the left inset of Fig. 8a, high-*Q* WGMs in a microtoroid are excited through a tapered fiber. To perform photothermal absorption spectroscopy, a second free-space beam is focused on the surface of the microresonator, where a single gold nanoparticle is deposited. The excitation of the gold nanoparticle gives rise to a local temperature increase due to photothermal absorption, which redshifts the resonance. Photothermal absorption spectroscopy can be achieved by tuning the pump laser wavelength. A PDH locking system can be applied to the probe laser to improve the thermal shift sensitivity down to a single attometer. It should be noted here that an all-glass microtoroid (the right inset of Fig. 8a) is used to perform the photothermal absorption spectroscopy experiment to reduce the photothermal background noise of the silicon pillar of a normal silica-on-silicon toroid in the visible region.

On the other hand, photothermal absorption imaging can also be achieved by scanning the pump beam across the surface of the microresonator, as shown in Fig. 8b, c^62–64^. In the experiment, mode shift signals are recorded in real time when scanning the pump laser beam spatially on the top surface of the resonators, which creates a thermal shift image. Experimentally, large-area thermal maps of a normal silica toroid on a silicon pillar (left) and an all-glass toroid (right) are recorded to compare the background noise (Fig. 8b). The background noise of the photothermal image for the all-glass microtoroid is much lower than that of the normal toroid on a silicon pillar at 630 nm, which represents a great advantage of the all-glass microtoroid resonator as a platform for photothermal imaging. Furthermore, individual gold nanorods with a size of 40 nm by 80 nm are deposited onto the all-glass microtoroid, which are then imaged by thermal shifts. Experimentally, large-area photothermal imaging is first utilized to find the position of the nanorod on the microtoroid (Fig. 8b). High-resolution thermal imaging is then performed to image individual nanorods, as shown in Fig. 8c, with an SEM image presented in the top inset.

## Thermal stabilization

The thermal stability of the WGM resonance is of great importance in almost all applications, such as lasing, optical sensing, optomechanics, nonlinear optics, and imaging. Various techniques have been developed to eliminate or reduce thermal noise to achieve thermal stabilization, which will be summarized in this section.

### Thermo-optic coefficient compensation

**Fig. 9:**
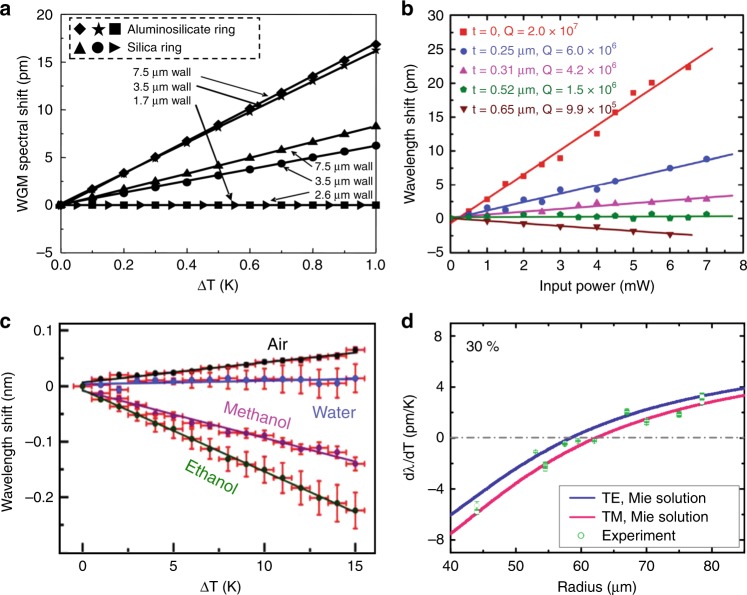
Thermo-optic compensation in WGM microresonators. **a** Thermal sensitivity reduction by optimizing the geometry of liquid core aluminosilicate and silica optical rings^129^. **b**–**d** Thermo-optic compensation of WGM microresonators by coating with PDMS (**b**)^131^, a quantum dot (**c**)^133^, and glycerol content in the surrounding aqueous medium (**d**)^134^. **a** Reprinted with permission from ref. ^129^ [OSA The Optical Society]. **b** Reprinted with permission from ref. ^131^ [American Institute of Physics]. **c** Reprinted with permission from ref. ^133^ [American Institute of Physics]. **d** Reprinted with permission from ref. ^134^ [American Institute of Physics]

Microresonators immune to thermal fluctuations have been fabricated by either optimizing the resonator structures/geometries or materials. For example, in a liquid core optical ring resonator, an optimization of the resonator’s geometry with respect to the surrounding medium can facilitate a reduction in the WGM’s thermal sensitivity. As shown in Fig. 9a, liquid core optical rings with a wall thickness of 1.7 μm for an aluminosilicate microring and 2.6 μm for a fused silica microring demonstrate minimum susceptibilities to temperature fluctuations^129^.

Hybrid microresonators fabricated by materials with opposite thermo-optic coefficients can also realize thermal stabilization. Theoretically, the thermal shift of WGMs in a silica microsphere with a positive thermo-optic coefficient could be compensated by coating a thin layer of a soft material with an opposite thermo-optic coefficient onto the surface of the microsphere^130^. He et al. demonstrated that a thin layer of PDMS coated on a silica microtoroid resonator could eliminate the thermal shift and broadening due to the thermo-optic compensation enabled by the opposite thermo-optic coefficients (Fig. 9b)^131^. Similar experiments were also performed with KD-310 glue^132^ and quantum dot^133^-coated microspheres (Fig. 9c). In addition, compensation of the thermal effect in a microsphere was also demonstrated by optimizing the glycerol content in the surrounding aqueous medium (Fig. 9d)^134^. The same stabilization scheme was utilized to characterize the thermal response of various polymer layers and protein molecules adsorbed to the resonator’s surface.

### Thermal locking stabilization technique

**Fig. 10:**
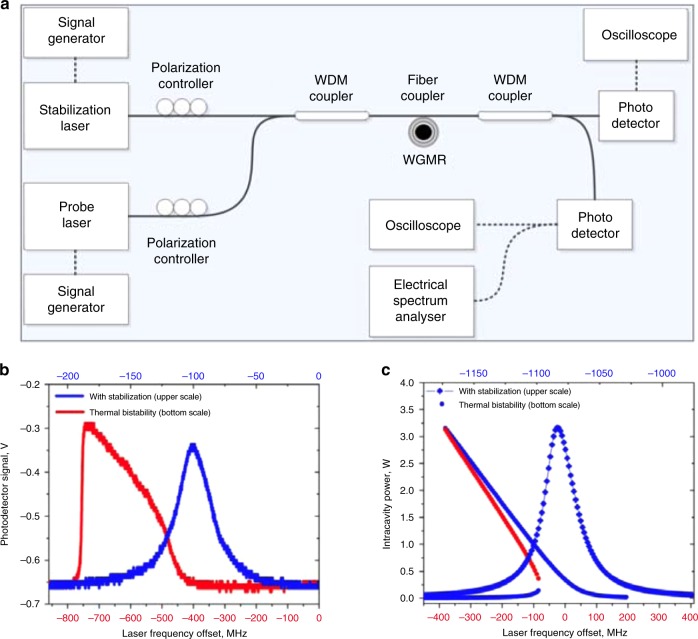
Thermal locking thermal stabilization technique. **a** Schematic of the thermal locking stabilization setup. **b** Experimental spectrum of a high-*Q* WGM with (blue) and without (red) thermal locking stabilization. **c** Simulation results of a high-*Q* WGM with (blue) and without (red) thermal locking stabilization^135^. Reprinted with permission from ref. ^135^ [OSA The Optical Society]

As mentioned previously, optothermal spectroscopy using thermal bistability has been adopted to eliminate the spectral perturbations caused by temperature fluctuations^57^. Here, we introduce another active thermal stabilization technique assisted by the thermal locking of an additional laser^135^. Two WGMs with different resonance wavelengths and similar mode volumes were exploited in this approach. As shown in Fig. 10a, two tunable lasers are used: one laser operates in the 1550 nm band as a pump laser, and the other laser operates in the 1450 nm band, serving as a probe laser. Two WDM couplers are utilized to combine the pump and probe lasers and then separate the transmitted signals. A fiber taper waveguide is used to couple light into WGMs of a microtoroid. In the experiment, the WGM in the 1450 nm wavelength band is first scanned and recorded; the thermal nonlinearity is shown as the red curve in Fig. 10b. Then, the pump laser at 1550 nm is applied and thermally locked to the linear region of the second high-*Q* mode in the 1550 nm wavelength band. Heating induced by the 1550 nm laser shifts the probe mode in the 1450 nm wavelength band. With the help of this thermal locking stabilization technique, the probe WGM in the 1450 nm wavelength band exhibits a perfect Lorentzian lineshape, as shown in Fig. 10b (blue curve). In addition, Fig. 10c shows the simulation results, which fit very well with the corresponding experimental data.

### Self-referenced dual-mode stabilization technique

Another scheme employed to stabilize the resonator temperature is the self-referenced dual-mode temperature stabilization technique based on a simultaneous readout of TE and TM WGMs. Specifically, the resonance frequencies of TM and TE WGMs in a microresonator have different temperature coefficients; thus, the self-referenced mode shift difference between the modes can serve as a perfect temperature sensing signal, which can also be utilized for active stabilization of the cavity temperature^136–139^.

**Fig. 11:**
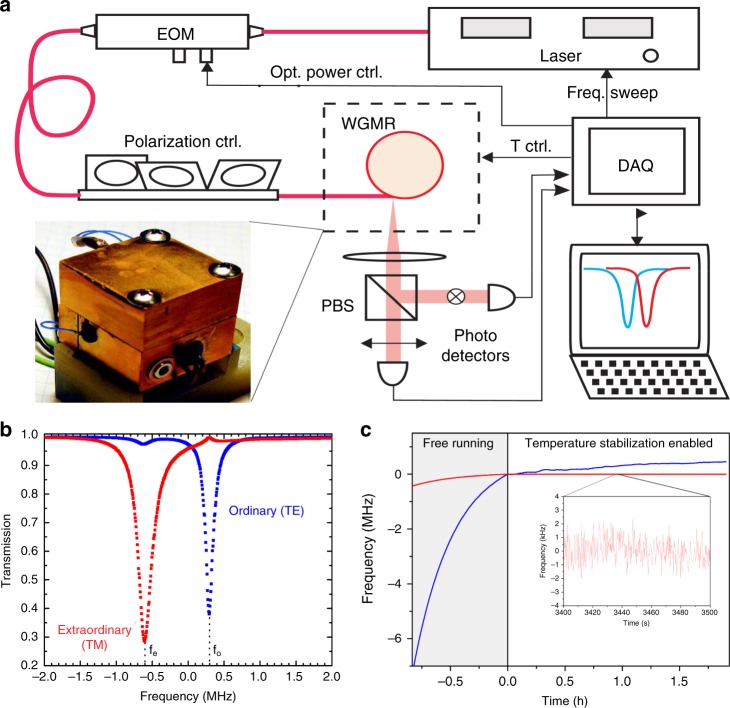
Self-referenced dual-mode thermal stabilization technique. **a** The experimental setup of the self-referenced dual-mode stabilization technique^136^. **b** A pair of TE and TM WGMs with a small frequency detuning Δ*f* = *f*_o_ − *f*_e_^136^. **c** The long-term absolute resonance frequency and dual-mode frequency are shown as blue and red curves, respectively, in the free-running case (*t* < 0 h) and the case with the temperature stabilized (*t* > 0 h) with the dual-mode technique. Inset: Magnification of the frequency fluctuation^137^. Reprinted with permission from ref. ^137^ [OSA The Optical Society]

Experimentally, a millimeter-sized MgF_2_ disk resonator is excited by an angle-polished fiber coupler. The TE and TM modes are controlled by a fiber-based polarization controller, split by a polarizing beam splitter (PBS), and then monitored by two photodetectors, as shown in Fig. 11a. A data acquisition card is used to collect the signals of the photodetectors, which also provides feedback to an electro-optic modulator (EOM) to achieve the self-referenced dual-mode stabilization process. A pair of TE and TM WGMs with a small frequency detuning are shown in Fig. 11b^136^. The average frequency of these two resonances is utilized to locate the center of the next frequency sweep. The difference Δ*f* = *f*_o_ − *f*_e_ serves as the feedback error signal for temperature stabilization. Two feedback loops are used to achieve the temperature stabilization. The heaters in the brass cube are employed for the first feedback loop for long-term stabilization. An amplitude EOM with a high-speed heating effect is used to adjust the optical power, which provides short-term stabilization. Temperature stabilization on the order of a few nano-Kelvin was achieved (Fig. 11c).

## Conclusion

In this review, we have briefly reviewed the mechanisms, techniques, and applications of thermo-optic dynamics in WGM microresonators. Thermal nonlinear effects, including both thermal bistability and thermal oscillation, have been discussed. Optothermal spectroscopy, thermal relaxation parameter measurement and optical thermal nonreciprocity in microresonators can be realized with the help of thermal bistability effects. Thermal tuning and thermal sensing are two examples of applications of the thermo-optic effect, which have been explored in various WGM resonator structures. By thermally scanning the resonance frequency of a microresonator, a single-frequency laser with much lower noise and narrower linewidth can be used to reduce the noise of a frequency comb and an optical soliton. In addition, thermal locking techniques and photothermal imaging implemented with WGM microresonators have also been reviewed. Finally, we have introduced some techniques to achieve thermal stabilization, which is a critical prerequisite in many high-performance WGM applications.

The research field of thermo-optic dynamics in WGMs continues to grow. Looking ahead, more technological development and new applications will benefit from discoveries in this particular area. For example, the PDH locking technique could be exploited for thermal sensing by monitoring the feedback voltage signal^140^. A fast thermal scanning technique may find applications in optomechanics, nonlinear optics, optical trapping, sensing and imaging. Thermal stabilization techniques hold great potential for ultrastable microlasers by suppressing temperature fluctuations. Finally, resonator-assisted photothermal absorption spectroscopy and imaging techniques will open up new avenues to study materials and structure properties at the nanoscale. It is also worth noting that more opportunities will arise when materials with diverse thermo-optic properties are utilized in the resonators. Similar physics and mechanisms and the associated technologies could be explored with other optical resonant structures.
